# The Evolving Field of Cardio-Rheumatology: Global Patterns and Research Gaps, 1953-2025

**DOI:** 10.7759/cureus.87901

**Published:** 2025-07-14

**Authors:** Olatokun Akano, Oluwaremilekun Tolu-Akinnawo, Kelechukwu P Ughagwu, Olumide Akinmoju, Selimat Ibrahim, Marvellous Oluwadare, Layal E Omaruddin, Oluwamisimi M Abib, Olamide Odusola, Toluwalase Awoyemi

**Affiliations:** 1 Neurological Surgery, Northwestern University Feinberg School of Medicine, Chicago, USA; 2 Neurological Surgery, University College Hospital, Ibadan, Ibadan, NGA; 3 Internal Medicine, Meharry Medical College, Nashville, USA; 4 Internal Medicine, Jefferson Einstein Philadelphia Hospital, Philadelphia, USA; 5 Internal Medicine, College of Medicine, University of Ibadan, Ibadan, NGA; 6 Family Medicine, EHA Clinics, Abuja, NGA; 7 Medicine and Surgery, Afe Babalola University, Ado-Ekiti, NGA; 8 Internal Medicine, Royal College of Surgeons in Ireland - Bahrain, Busaiteen, BHR; 9 Internal Medicine, Piedmont Athens Regional Medical Center, Athens, USA; 10 Medicine and Surgery, College of Medicine, University of Ibadan, Ibadan, NGA; 11 Medicine, Northwestern University Feinberg School of Medicine, Chicago, USA

**Keywords:** autoimmune diseases, bibliometric analyses, cardio-rheumatology, cardiovascular diseases, global patterns, rheumatology & autoimmune diseases

## Abstract

Cardio-rheumatology, a rapidly evolving field at the intersection of cardiovascular and autoimmune diseases, remains intellectually fragmented despite increasing clinical and research interest driven by the rising burden of cardiovascular complications in rheumatic conditions. This was a bibliometric analysis of 3,709 articles from 1953 to March 2025, identified via Scopus databases, that assessed publication trends, citation metrics, authorship patterns, and global contributions, revealing a sharp rise in output post 2010, with the United States leading in both volume and impact. However, international collaboration was limited (11.6% multi-country authorship), and productivity was concentrated among a few contributors. Dominant themes included myocarditis, pericarditis, and accelerated atherosclerosis, while emerging areas such as cardiac sarcoidosis, advanced imaging, and biologic therapy-associated cardiotoxicity are gaining prominence. This analysis outlines the foundational structure of cardio-rheumatology and underscores the need for greater interdisciplinary and international integration to advance the field and improve patient outcomes.

## Introduction and background

Cardio-rheumatology is an emerging subspecialty that is focused on the intersection of cardiovascular disease and rheumatic diseases, particularly their inflammatory effect on the heart. Cardiovascular involvement in systemic rheumatic and autoimmune diseases is a major contributor to excess morbidity and mortality, with conditions such as rheumatoid arthritis, systemic lupus erythematosus, and sarcoidosis associated with a wide array of cardiovascular complications, including pericarditis, myocarditis, cardiomyopathy, and accelerated atherosclerosis [1-3]. While these associations are gaining increasing recognition in clinical and research contexts, the interdisciplinary domain of cardio-rheumatology remains underdeveloped. In contrast to established hybrid fields like cardio-oncology, cardio-rheumatology lacks formal structural cohesion, dedicated training pathways, and consensus frameworks for clinical care and research integration.

Recent advances in immunopathology, cardiovascular imaging, and targeted therapeutics have renewed attention to the cardiac manifestations of systemic inflammatory diseases. The advent of biologics and immune-modulating agents has broadened the clinical phenotype, revealing novel cardiotoxicities and complex immune-mediated mechanisms of cardiac injury [4]. These evolving challenges have necessitated more integrated care models, fostering collaboration between cardiology and rheumatology. As a result, a new wave of research is emerging that seeks to characterize, diagnose, and manage inflammatory cardiovascular syndromes with increasing precision.

Despite this growing clinical and academic interest, the scholarly evolution of cardio-rheumatology has not been formally quantified. Bibliometric analysis, a methodological approach for assessing the structure, dynamics, and impact of scientific disciplines, offers a valuable lens to evaluate the field’s growth trajectory, thematic distribution, and collaborative landscape [5]. This approach has successfully been used to define the maturation and influence of related hybrid disciplines such as cardio-oncology. It holds similar promise for elucidating the current status and future direction of cardio-rheumatology. This bibliometric analysis outlines the foundational structure of cardio-rheumatology and underscores the need for greater interdisciplinary and international integration to advance the field and improve patient outcomes.

## Review

Material and methods

Data Sources and Search Strategy

A comprehensive bibliometric dataset was compiled from the Scopus database on March 27, 2025, covering the period from 1953 till the date of search. The query strategy combined free-text and controlled vocabulary terms targeting the TITLE field, with search terms encompassing major concepts in cardio-rheumatology. These included but were not limited to: “Cardio-rheumatology”, “Autoimmune heart disease”, “Inflammatory cardiomyopathy”, “Autoimmune myocarditis”, “Cardiac sarcoidosis”, and related terms. The complete search syntax is available in the supplementary materials. Records were downloaded in BibTeX format and analyzed using the Bibliometrix R package v4.3.0 (K-Synth Srl, Naples, Italy). The extracted metadata fields included authorship, institutional affiliations, geographic origin, journal sources, keywords, citation counts, and reference data. Duplicate entries were systematically identified and excluded before downstream analyses (which refers to the subsequent steps taken after initial data collection).

Bibliometric and Network Analyses

Descriptive bibliometric indicators, including total publication output, annual growth rate, and mean citation count per document, were computed. Publications were classified into standard categories: original research articles, reviews, conference abstracts, editorials, and book chapters. At the author level, metrics included total output, fractional publication contribution, and citation-based impact measures. International collaboration was evaluated using the multiple country publication (MCP) ratio, the proportion of co-authored papers involving authors from more than one country. Keyword-based thematic mapping was performed via co-word analysis, using author-defined keywords to generate a co-occurrence network that delineated the intellectual structure of the field. Structural properties of the network, such as density, transitivity, diameter, mean path length, and degree centralization, were quantified. Thematic clusters were plotted along two axes: centrality (reflecting relevance) and density (indicating internal cohesion), yielding a quadrant-based thematic map comprising motor themes, basic themes, niche themes, and emerging or declining areas.

Collaboration Network Construction

Three types of collaboration structures were analyzed: author-level, institution-level, and international collaborations. Author collaboration networks were constructed from co-authorship relationships, enabling the identification of influential research clusters and collaborative subgroups. Institutional collaboration was assessed by evaluating cross-institutional co-authorships, shedding light on academic and clinical partnerships.

Global collaboration patterns were quantified using the MCP ratio. A three-field plot was constructed to visualize interconnected structures across keywords, authors, and countries, with connection strengths weighted by the frequency of co-occurrence. All data analyses were performed in R (v4.3.0) using Bibliometrix, with supplemental visualizations generated using the graph (https://igraph.org/) and ggplot2 (https://ggplot2.tidyverse.org/) libraries. 

Results

Document Characteristics and Authorship Structure

Between 1953 and 2025, 3,709 documents were published across 775 sources, reflecting an annual growth rate of 5.55%. The most notable increase occurred post 2015, reaching a peak in 2022 with 289 publications, the highest yearly output. Although there was a slight decline in 2023 (268) and 2024 (272), publication volume remained robust. The dataset comprised a variety of document types, predominantly original research articles (1,731), meeting abstracts (1,071), reviews (290), and editorial materials (276), along with letters, book chapters, and notes. The average document age was 11.1 years, with an average of 13.99 total citations and 1.264 citations per year. The dataset also included 5,312 KeyWords Plus and 2,794 author-provided keywords, indicating a broad thematic range (Table 1).

**Table 1:** Main bibliometric indicators

Category	Indicator	Value
General Info	Timespan	1953–2025
	Sources (Journals, Books, etc.), n	775
	Documents, n	3,709
	Annual Growth Rate, %	5.55
	Document Average Age (years)	11.1
	Average Citations per Document	13.99
	Average Citations per Year per Document	1.264
Content	KeyWords Plus, n	5,312
	Author-Given Keywords, n	2,794
Authorship	Total Authors, n	12,859
	Author Appearances, n	26,265
	Authors of Single-Author Documents, n	126
Collaboration Metrics	Single-Author Documents, n	156
	Documents per Author	0.288
	Co-Authors per Document	7.08
	International Co-Authorships (%)	10.38

Most Relevant Sources

**Figure 1:**
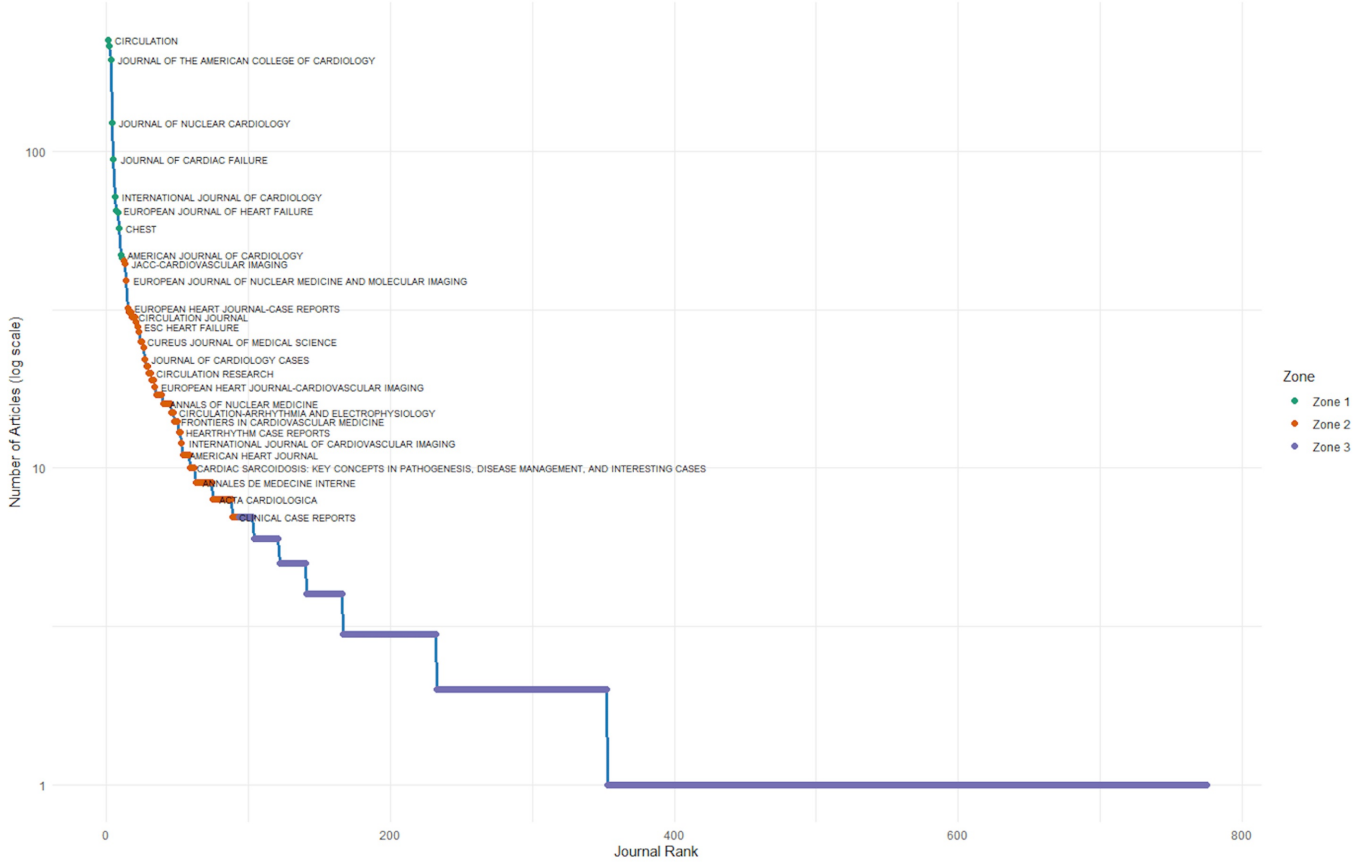
Application of Bradford’s Law to cardio-rheumatology literature, dividing sources into three zones based on citation productivity. Zone 1 includes a small core of highly productive journals; Zones 2 and 3 comprise an increasing number of journals with progressively fewer citations.

Bradford's analysis reveals a core-periphery structure in the distribution of literature sources on cardio-rheumatology (Figure 1).

Zone 1 contains the most influential journals with the highest concentration of articles and citations, including *Circulation* (224 articles, 3,132 citations), *European Heart Journal* (214 articles), and *Journal of the American College of Cardiology* (194 articles). These three journals account for a substantial share of the field’s high-impact literature. This indicates that Bradford's Law holds in this dataset: a small nucleus of journals contributes disproportionately to the citation impact, while productivity and influence taper off across Zones 2 and 3. Table 2 contains the most relevant sources.

**Table 2:** Most relevant sources by article count

Rank	Source	Articles
1	Circulation	224
2	European Heart Journal	214
3	Journal of the American College of Cardiology	194
4	Journal of Nuclear Cardiology	123
5	Journal of Cardiac Failure	94
6	International Journal of Cardiology	72
7	European Journal of Heart Failure	65
8	Journal of Nuclear Medicine	64
9	Chest	57
10	American Journal of Cardiology	47

Authorship and Collaboration Patterns

A total of 12,859 unique authors contributed to the body of literature, with 26,265 total author appearances, reflecting an intensely collaborative research environment. Only 156 documents were single-authored, with just 126 authors producing single-author publications. The field showed substantial teamwork, averaging 7.08 co-authors per paper, and 10.38% of publications involved international co-authorship (Table 1). These figures underscore a globally distributed, team-oriented model of scholarship. 

**Figure 2:**
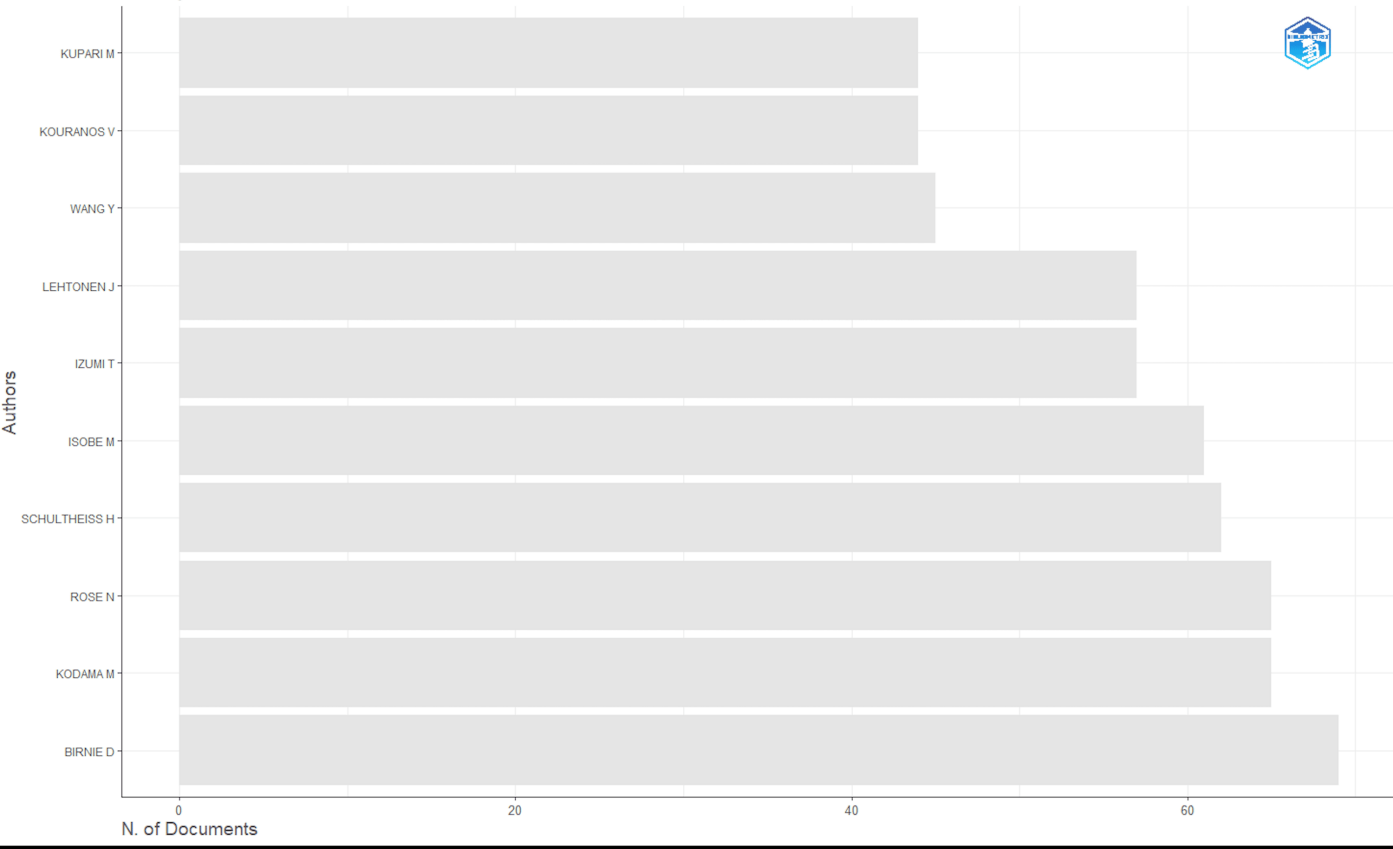
Most productive authors This is a bar chart of the top authors in cardio-rheumatology ranked by the number of published documents, highlighting leading contributors to the field. Image created with Bibliometrix (K-Synth Srl, Naples, Italy)

**Figure 3:**
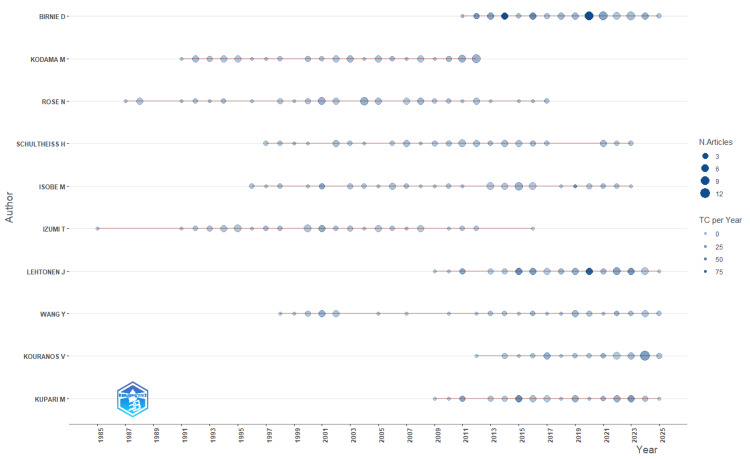
Temporal distribution of the top authors’ publications from 1985 to 2023 Bubble size represents the number of articles per year and color intensity denotes the average citation rate (total citations/year), capturing both productivity and impact over time. Image created with Bibliometrix (K-Synth Srl, Naples, Italy)

The most prolific contributor was Birnie D, leading in total publications (69) and fractionalized authorship (14.84), indicating sustained engagement and centrality within the field. Other top contributors included Kodama M (65 publications), Rose N (65), and Schultheiss H (62), all of whom also ranked among the top 10 for fractionalized authorship (Figures 2, 3). Table 3 shows the most productive authors.

**Table 3:** Most productive authors: full vs. fractionalized counts

Rank	Author (Full Count)	Articles	Author (Fractionalized)	Articles (Fractionalized)
1	Birnie D	69	Birnie D	14.84
2	Kodama M	65	Rose N	13.14
3	Rose N	65	Schultheiss H	12.84
4	Schultheiss H	62	Lehtonen J	11.81
5	Isobe M	61	Kupari M	11.03
6	Izumi T	57	Isobe M	9.96
7	Lehtonen J	57	Izumi T	9.47
8	Wang Y	45	Kodama M	8.12
9	Kouranos V	44	Blankstein R	7.46
10	Kupari M	44	Wang Y	7.41

Institutional Collaboration and Citation Impact

The most highly cited works in cardio-rheumatology underscore foundational and recent advances in inflammatory and autoimmune cardiovascular conditions. Leading the citation rankings is Birnie et al., 2014 (*Heart Rhythm*) [6], with 989 citations, offering a key clinical reference on cardiac sarcoidosis. Tschoepe et al., 2021 (*Nature*) [7] stands out for its remarkable citation rate (139.8 citations/year) and highest normalized citation score (NTC) of 72.1, signaling a significant recent contribution, likely due to its updated conceptual framework for myocarditis and inflammatory cardiomyopathy (Table 4).

**Table 4:** Most cited papers TC: total citation: NCS: normalized citation score

Rank	Authors	Journal (Year)	Article Title	TC	TC/Year	NTC
1	Birnie et al. [6]	*Heart Rhythm* (2014)	HRS expert consensus statement on the diagnosis and management of arrhythmias associated with cardiac sarcoidosis	989	82.4	36.3
2	Tschoepe et al. [7]	*Nat Rev Cardiol *(2021)	Myocarditis and inflammatory cardiomyopathy: current evidence and future directions	699	139.8	72.1
3	Vakhrusheva et al. [8]	*Circ Res* (2008)	Sirt7 increases stress resistance of cardiomyocytes and prevents apoptosis and inflammatory cardiomyopathy in mice	504	28.0	22.0
4	Blankstein et al. [9]	*J Am Coll Cardiol* (2014)	Cardiac positron emission tomography enhances prognostic assessments of patients with suspected cardiac sarcoidosis	493	41.1	18.1
5	Frustaci et al. [10]	*Eur Heart J* (2009)	Randomized study on the efficacy of immunosuppressive therapy in patients with virus-negative inflammatory cardiomyopathy: the TIMIC study	491	28.9	19.9
6	Yazaki et al. [11]	*Am J Cardiol *(2001)	Prognostic determinants of long-term survival in Japanese patients with cardiac sarcoidosis treated with prednisone	485	19.4	13.4
7	Ammirati et al. [12]	*Circ Heart Fail* (2020)	Management of acute myocarditis and chronic inflammatory cardiomyopathy: an expert consensus document	458	76.3	54.7
8	Youssef et al. [13]	*J Nucl Med* (2012)	The use of 18F-FDG PET in the diagnosis of cardiac sarcoidosis: a systematic review and meta-analysis including the Ontario experience	440	31.4	20.0
9	Smedema et al. [14]	*J Am Coll Cardiol *(2005)	Evaluation of the accuracy of gadolinium-enhanced cardiovascular magnetic resonance in the diagnosis of cardiac sarcoidosis	412	19.6	14.4
10	Kandolin et al. [15]	*Circulation* (2015)	Cardiac sarcoidosis: epidemiology, characteristics, and outcome over 25 years in a nationwide study	399	36.3	29.8

**Figure 4:**
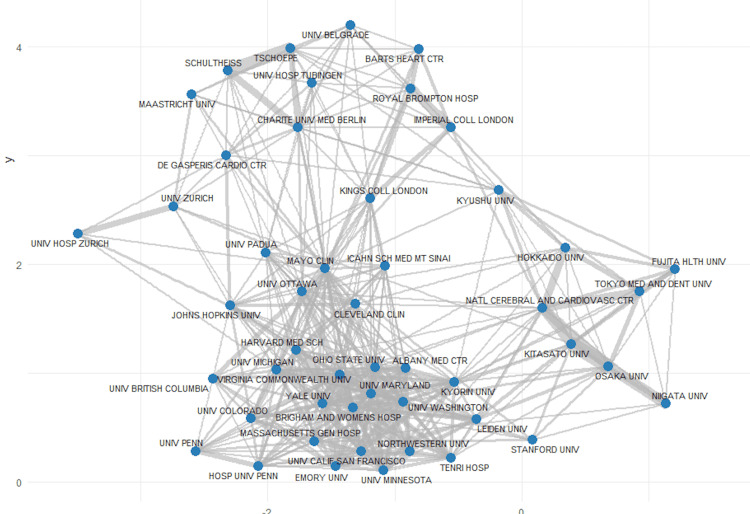
University collaboration network in cardio-rheumatology Each node represents a university, with edge thickness reflecting the strength of co-authorship ties. Prominent clusters reveal strong inter-institutional partnerships, with leading universities positioned centrally as key bridges within the global research network.

The institutional collaboration map revealed distinct, interconnected clusters, highlighting robust partnerships across institutions that likely enhance research visibility, knowledge exchange, and overall scientific impact (Figure 4).

Geographic Distribution and Collaboration Patterns

The United States and Japan were the most prolific contributors to cardio-rheumatology research, representing 22.59% (838 publications) and 18.41% (683 publications) of corresponding author affiliations. They also ranked highest in total citations (United States: 14,262; Japan: 12,111). However, smaller countries such as Finland (39.18), Switzerland (37.46), and Canada (34.45) exhibited the highest citation impact per publication, reflecting a focus on high-quality research outputs (Table 5).

**Table 5:** Total citations per country United States and Japan lead in total citations due to their high publication volume, contributing the largest citation shares globally. Finland, Switzerland, and Canada show exceptionally high per-article impact (39.18, 37.46, and 34.45 citations/article, respectively), highlighting fewer but high-quality or influential publications.

Rank	Country	Total Citations	Average Citations per Article
1	United States	14,262	17.02
2	Japan	12,111	17.73
3	Germany	4,849	24.61
4	Canada	4,100	34.45
5	Switzerland	2,135	37.46
6	China	2,057	12.94
7	Italy	1,739	20.95
8	Finland	1,489	39.18
9	United Kingdom	1,031	11.58
10	France	895	14.92

**Figure 5:**
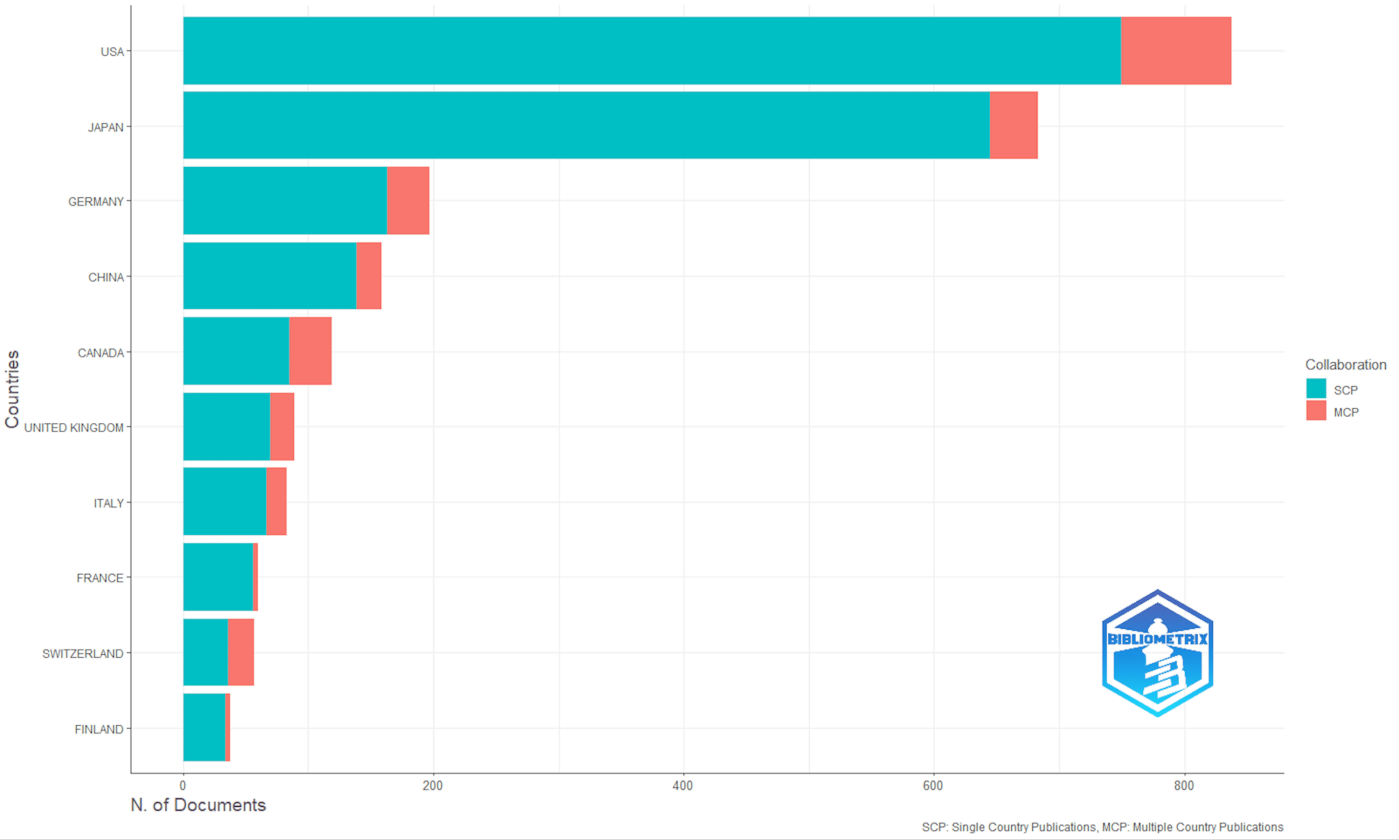
Top contributing countries to cardio-rheumatology research Bars are disaggregated by collaboration type: SCP (single-country publications) and MCP (multi-country publications). Image created with Bibliometrix (K-Synth Srl, Naples, Italy)

**Figure 6:**
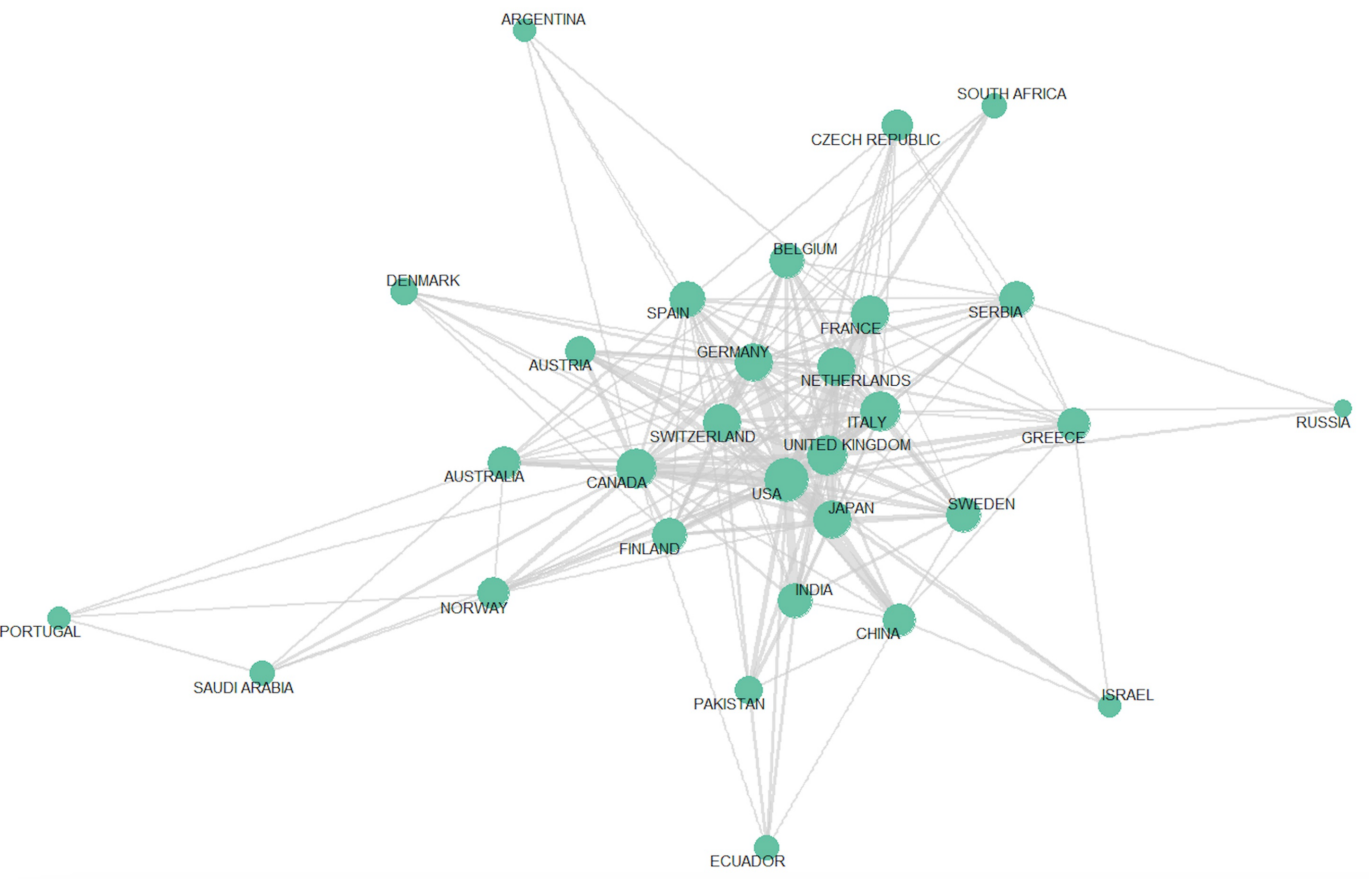
International country collaborative network Nodes represent countries and edge thickness reflects the strength of co-authorship links, highlighting global research connectivity.

Switzerland recorded the highest MCP ratio at 36.8%, followed by Canada at 28.6%, underscoring strong international collaboration despite fewer overall publications. These trends suggest that while research volume is centered in a few countries, cross-border collaborations, particularly across Europe and North America, substantially shape the global cardio-rheumatology research agenda. Conversely, Japan and France demonstrated lower MCP ratios (5.56% and 6.67%, respectively), indicating a greater emphasis on nationally driven research (Figures 5, 6). Table 6 shows the country collaboration metrics.

**Table 6:** Corresponding author country and colloborations SCP: single-country publications; MCP: multi-country publications

Rank	Country	Articles	Frequency	SCP	MCP	MCP Ratio
1	United States	838	0.3083	750	88	0.1050
2	Japan	683	0.2513	645	38	0.0556
3	Germany	197	0.0725	163	34	0.1726
4	China	159	0.0585	139	20	0.1258
5	Canada	119	0.0438	85	34	0.2857
6	United Kingdom	89	0.0327	70	19	0.2135
7	Italy	83	0.0305	67	16	0.1928
8	France	60	0.0221	56	4	0.0667
9	Switzerland	57	0.0210	36	21	0.3684
10	Finland	38	0.0140	34	4	0.1053

**Figure 7:**
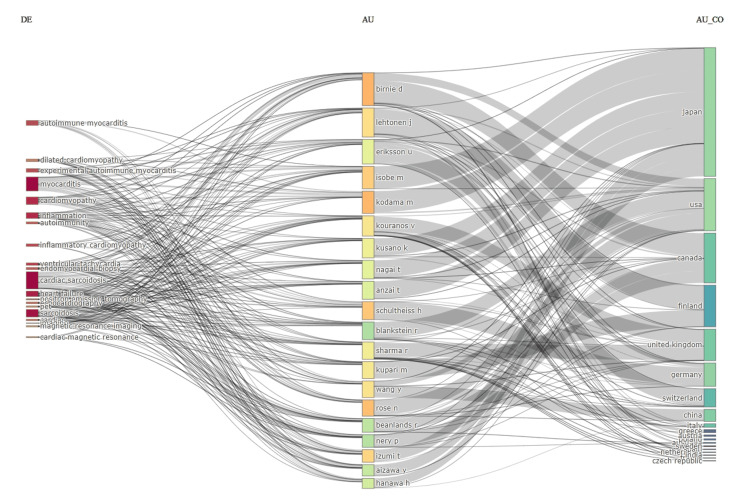
Sankey diagram (three-field plot) illustrates the thematic connections across three dimensions: keywords (DE), authors (AU), and countries (AU_CO) Flow width represents the volume of publications associated with each link, highlighting the most studied topics, prolific researchers, and their geographic distribution.

The top researchers in the field were affiliated with institutions in Japan, the United States, Canada, Finland, and Germany. For instance, Birnie D and Blankstein R were based in Canada and the United States, respectively; Lehtonen J and Kupari M were affiliated with Finnish institutions. Prominent Japanese contributors included Isobe M, Kodama M, and Kusano K. Schultheiss H represented Germany. Additional key contributions came from institutions in Switzerland, China, the United Kingdom, and Italy, reflecting a globally distributed yet topically aligned research network (Figure 7).

Research Topics and Core Themes

The most widely studied topics in cardio-rheumatology included cardiac sarcoidosis (705 articles), myocarditis (366), sarcoidosis (327), and heart failure (159), highlighting a strong emphasis on immune-mediated myocardial inflammation and its clinical implications (Table 7).

**Table 7:** Most relevant keywords Author keywords emphasize disease-specific and mechanistic terms (e.g., autoimmune myocarditis, ventricular tachycardia), reflecting intentional topic framing by researchers. KeyWord Plus captures broader biomedical descriptors (e.g., human, male, adult) indicative of demographic or structural metadata. Cardiac sarcoidosis is the dominant term in both keyword sets, confirming its centrality in the field.

Rank	Author Keywords	Articles	KeyWord Plus	Articles
1	Cardiac Sarcoidosis	705	Sarcoidosis	716
2	Myocarditis	366	Human	585
3	Sarcoidosis	327	Male	477
4	Heart Failure	169	Article	447
5	Inflammation	159	Female	447
6	Cardiomyopathy	152	Adult	426
7	Ventricular Tachycardia	101	Myocarditis	425
8	Inflammatory Cardiomyopathy	87	Humans	307
9	Autoimmune Myocarditis	76	Middle Aged	307
10	Experimental Autoimmune Myocarditis	73	Cardiomyopathy	302

**Figure 8:**
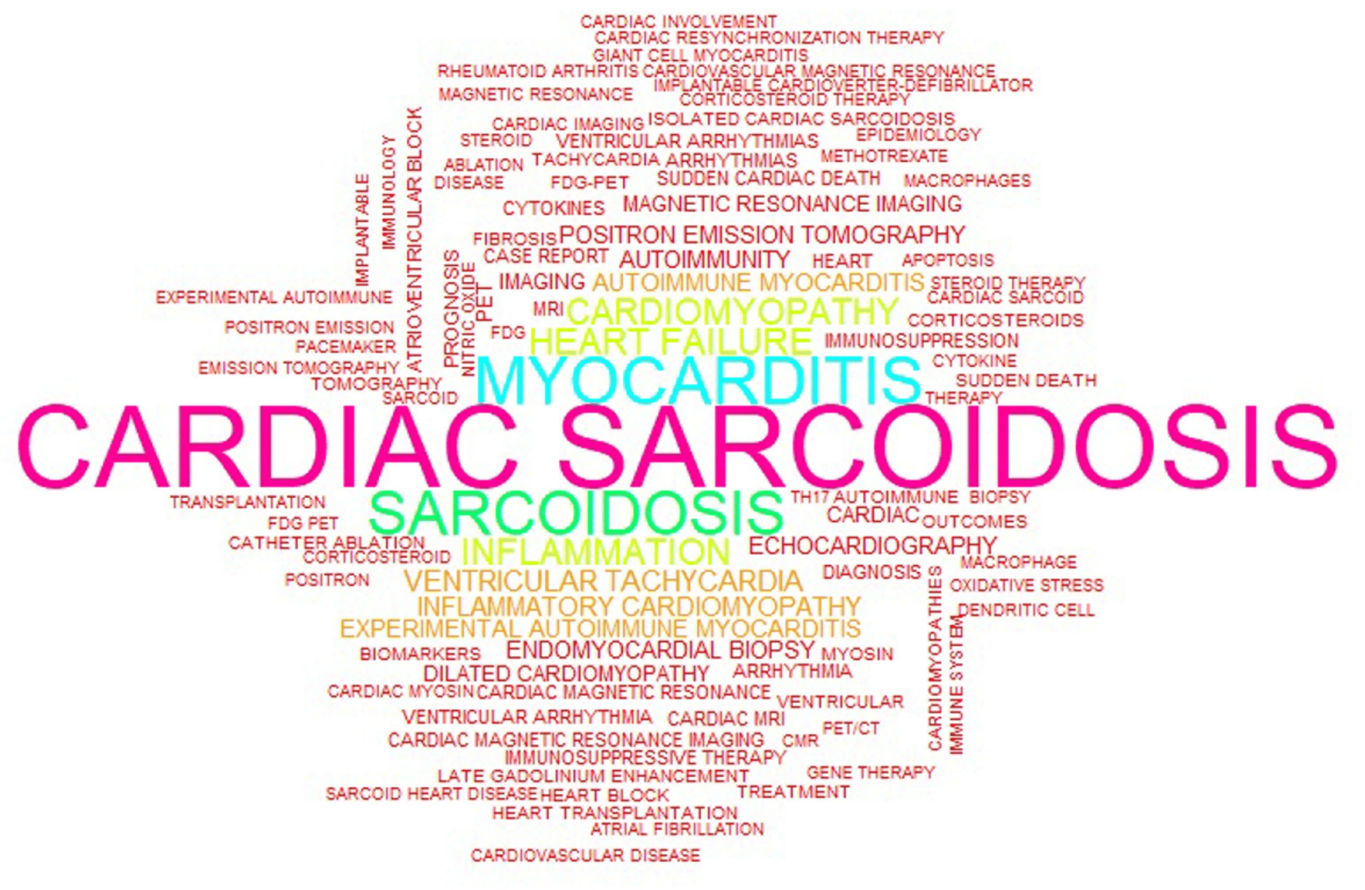
Word cloud of the most frequently used author keywords in cardio-rheumatology research The size of each term reflects its frequency, with dominant keywords such as myocarditis, cardiac sarcoidosis, and inflammation appearing most prominently

These themes were frequently featured as author keywords, indicating a deliberate focus within the literature. Leading researchers, such as Birnie D, Blankstein R, and Isobe M, were closely linked to these areas, particularly autoimmune myocarditis, inflammatory cardiomyopathy, cardiac sarcoidosis, and advanced cardiac imaging. Their consistent association with these topics underscores their significant impact and sustained influence in cardio-rheumatology (Figure 8).

Author Collaboration Network

The author collaboration network consisted of 12,859 unique nodes, representing individual researchers. The network was characterized by a low density of 0.001, indicating sparse overall connectivity among authors. Despite this, the transitivity coefficient was 0.413, suggesting moderate local clustering, where authors within subgroups tended to co-author. The maximum distance between any two connected nodes (diameter) was 13, while the average path length was 4.637, implying that most authors were separated by fewer than five degrees of collaboration. The degree of centralization was low (0.023), reflecting a decentralized structure without prominent hub authors.

Visual network inspection confirmed these metrics, revealing a fragmented topology of numerous loosely connected clusters. The distribution of nodes was diffuse, with no dominant central component. Clusters varied in size and were spatially dispersed, indicating that collaboration within the field remains primarily compartmentalized. No large-scale, cohesive core was evident, and inter-cluster links were limited. The structural attributes and visual configuration of the network directly support these observations.

Keyword Co-Occurrence Network

**Figure 9:**
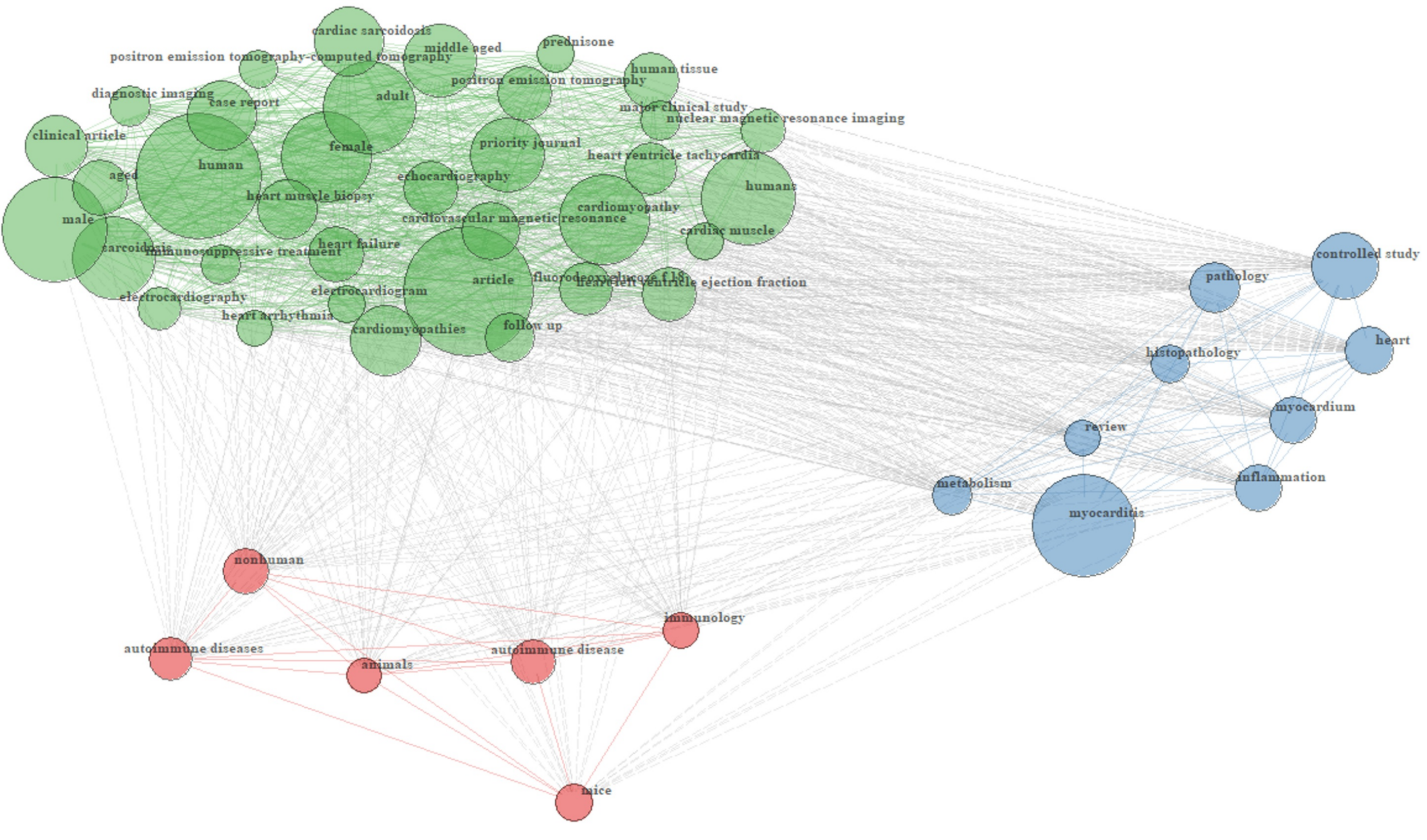
Co-occurrence network of author keywords in cardio-rheumatology research Nodes represent keywords, and edge thickness reflects the frequency of co-occurrence. The network reveals a densely connected core centered on clinical and diagnostic concepts, with prominent clusters of imaging modalities and frequent co-occurrence of demographic and biomedical descriptors.

The keyword co-occurrence network comprised 5,312 nodes, representing distinct terms used across the corpus. The network exhibited a density of 0.015 and transitivity of 0.214, indicating a sparsely connected yet moderately clustered structure. The maximum path length between any two nodes (diameter) was 5, and the average path length was 2.399, suggesting close semantic proximity across most keyword pairs. The degree of centralization was 0.531, reflecting a semi-centralized topology dominated by a subset of frequently co-occurring terms. Visual analysis revealed a densely connected core of clinical and diagnostic concepts. Keywords such as sarcoidosis, myocarditis, cardiomyopathy, cardiac sarcoidosis, heart failure, and echocardiography occupied central positions within the network. A prominent cluster of imaging-related terms was also evident, including positron emission tomography (PET), fluorodeoxyglucose (F18), cardiovascular magnetic resonance, and electrocardiography. Common demographic descriptors (male, female, adult, middle-aged, aged) and biomedical categories (inflammation, autoimmune disease, histopathology, metabolism) were frequently linked to clinical terms (Figure 9). The network structure highlights a research landscape focused on the diagnostic imaging, pathophysiology, and immunologic underpinnings of cardiac sarcoidosis.

Thematic Mapping of Cardio-Rheumatology Research

**Figure 10:**
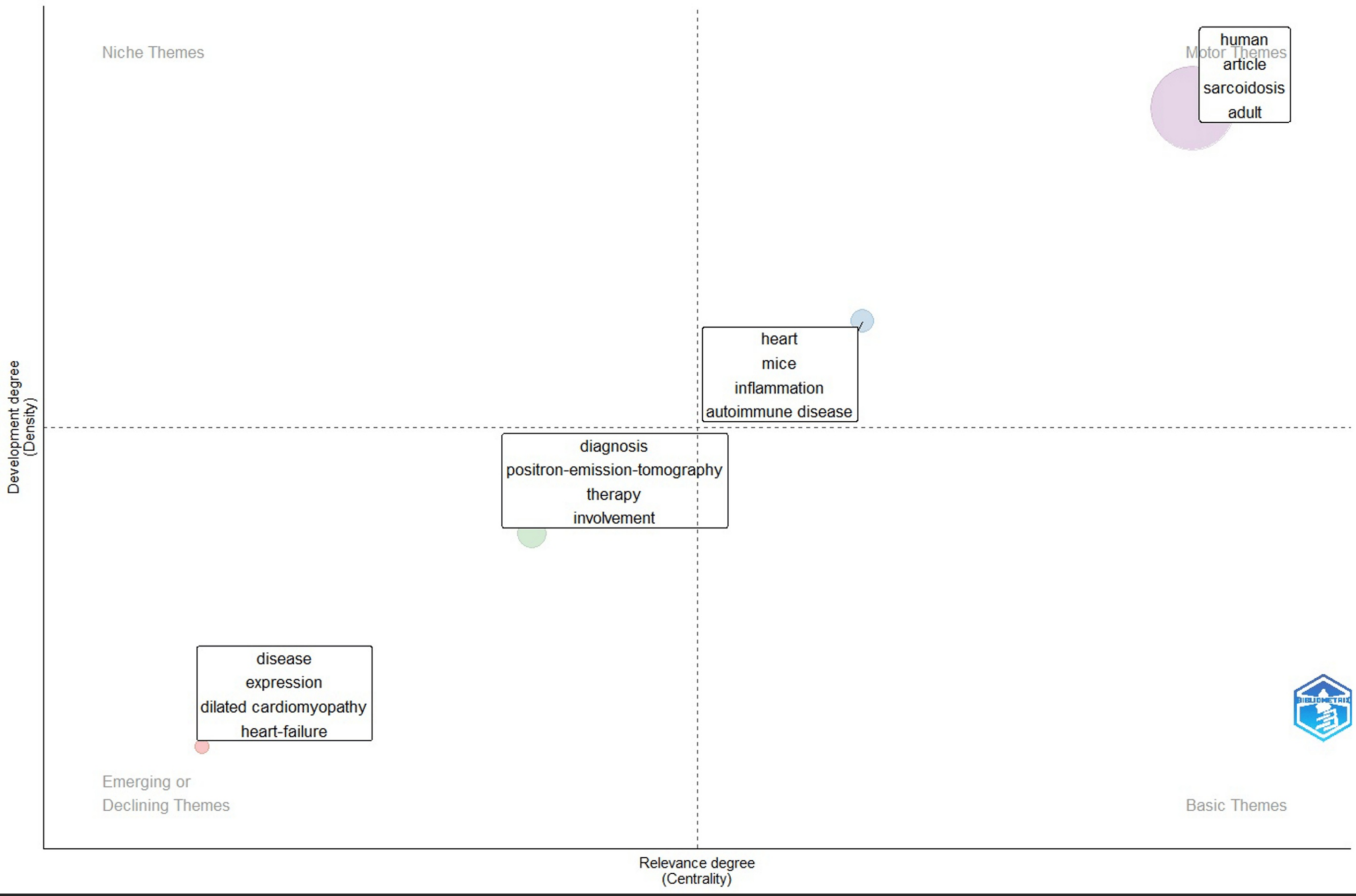
Thematic map of cardio-rheumatology research The map identifies major thematic areas within cardio-rheumatology, positioned according to their centrality (relevance to the field) and density (development). Themes in the upper-right quadrant represent well-developed and central "motor themes," while those in the lower-right reflect fundamental but less developed "basic themes." The upper-left quadrant includes specialized or "niche themes," and the lower-left highlights "emerging or declining themes." Image created with Bibliometrix (K-Synth Srl, Naples, Italy)

The thematic map (Figure 10) revealed a structured distribution of research foci across four quadrants defined by centrality (relevance) and density (development). The lower-right quadrant, corresponding to motor themes, included sarcoidosis, autoimmune disease, inflammation, and PET. These themes exhibited high centrality and density, indicating they are well-developed and integral to the research field. In the upper-right quadrant, representing niche themes, therapy, involvement, and diagnosis were positioned. These topics demonstrated strong internal development but lower connectivity to the broader field, suggesting specialized but mature subdomains. The basic themes in the lower-left quadrant included heart failure, dilated cardiomyopathy, heart, and human. These terms had high centrality but low density, reflecting foundational but less specialized areas of research that underpin multiple thematic areas. Finally, the upper-left quadrant, indicative of emerging or declining themes, contained mice, adult, expression, and article. These terms showed relatively low centrality and density, suggesting limited integration or declining prominence within the field’s current research trajectory. The overall structure highlights a domain centered on the pathophysiology and diagnostic imaging of sarcoidosis and autoimmune cardiac involvement, supported by foundational heart failure research and specialized diagnostic modalities.

Discussion

This bibliometric analysis reveals a steady growth in cardio-rheumatology research over the past 70 years, with a notable surge in publications since the early 2000s. The United States and Japan dominate the field, accounting for over half of the global output and citation impact. Major general cardiology journals such as *Circulation* and the *European Heart Journal* serve as key dissemination platforms. In contrast, specialized journals in nuclear cardiology and inflammation contribute disproportionately to the field’s intellectual development. Thematic and keyword analyses show that cardiac sarcoidosis, myocarditis, and rheumatic heart disease remain central, while emerging focus areas such as cardiac amyloidosis, immune-mediated myocardial inflammation, and multimodality imaging highlight the field’s growing complexity and interdisciplinary evolution.

Although cardio-rheumatology has experienced a notable expansion in scholarly output, our findings reveal a persistent deficiency in international collaboration, with only 10.38% of publications involving multi-country authorship. This limited global engagement contrasts sharply with the transnational nature of autoimmune and systemic inflammatory diseases, which affect populations across diverse geographic and socioeconomic contexts [16]. Countries such as Switzerland (MCP ratio: 36.8%), Canada (28.6%), and the United Kingdom (21.4%) demonstrate relatively strong collaborative networks, reflecting established infrastructures for cross-border academic partnerships. However, other prolific contributors, notably Japan (5.6%) and China (12.6%), exhibit markedly lower rates of international co-authorship, underscoring a structural imbalance in the global distribution of collaborative research efforts.

This imbalance is especially concerning given the disproportionate burden of rheumatic and inflammatory cardiovascular diseases in low- and middle-income countries (LMICs), where healthcare systems often face limited capacity for early detection, risk stratification, and disease-modifying interventions [17,18]. The underrepresentation of LMICs in collaborative cardio-rheumatology research reflects a missed scientific opportunity and a broader challenge in ensuring the global equity and relevance of emerging research agendas. Integrating LMIC perspectives and contexts into the research enterprise is essential for developing adaptable, scalable, and culturally informed global strategies to manage inflammatory cardiovascular disease [19]. Future initiatives should prioritize inclusive research networks, capacity-building partnerships, and funding mechanisms to generate equitable knowledge across geographic and economic divides.

The concentration of authorship and citation influence among a limited group of researchers, notably Birnie, Tschöpe, and Okura, reflects the formative structure of the cardio-rheumatology field. These individuals have produced consistently high-impact contributions, particularly in cardiac sarcoidosis, inflammatory cardiomyopathies, and the application of multimodal imaging modalities. Their recurrent appearance in the most-cited publications suggests the emergence of intellectual hubs that anchor the field’s knowledge base. While this concentration may signify strong leadership and expertise, it highlights limited academic diversification and underdeveloped mentorship pipelines that often characterize emerging disciplines [20]. Such centralization underscores the need for strategies that promote broader participation, including interdisciplinary training and targeted support for early-career investigators.

Co-occurrence and thematic mapping analyses further illuminate the intellectual structure of the field. Dominant keywords such as “inflammation,” “cardiomyopathy,” “myocarditis,” and “sarcoidosis” remain central, indicating that while the field is expanding, key mechanistic and diagnostic challenges persist. The positioning of cardiac sarcoidosis and myocarditis within the “motor themes” quadrant of the thematic map denotes their established clinical and research significance. Conversely, emerging topics such as cardiac amyloidosis, interleukin-6 (IL-6)-mediated myocardial injury, and autoimmune vasculitis are situated within the “niche themes” quadrant, reflecting their increasing relevance and capacity to shape future research directions. These thematic shifts are consistent with broader paradigms in precision medicine, where the convergence of immunology and cardiology drives a more granular understanding of inflammatory cardiovascular diseases [21,22]. Continued thematic diversification, supported by robust translational and clinical inquiry, will be essential to advancing the field toward a more mature and inclusive scientific identity.

Nonetheless, key structural and conceptual gaps remain. Chief among these is the absence of cohesive theoretical frameworks and standardized nomenclature, which hinders consistent diagnosis, classification, and management of immune-mediated cardiovascular conditions across institutions and specialties. This lack of consensus poses a significant barrier to developing universally applicable clinical guidelines and impedes the scalability of translational research efforts. Furthermore, the relatively modest citation density (13.99 citations per document) and elevated mean document age (11.1 years) suggest a continued reliance on legacy literature, with limited integration of recent advances in immunocardiology, precision medicine, and molecular diagnostics. While the absence of referenced citations in the dataset may partly reflect limitations of Scopus export formats, it may also underscore inconsistencies in citation practices across journals and disciplines, further complicating bibliometric assessment and knowledge dissemination.

Addressing these challenges will require deliberate efforts to foster international and interdisciplinary collaboration. Strengthening partnerships with LMICs, promoting equitable access to research infrastructure, and encouraging global data sharing are essential to ensuring that cardio-rheumatology research reflects diverse clinical contexts and informs universally relevant strategies. Establishing multicenter registries, standardized diagnostic frameworks, and collaborative networks can accelerate evidence generation and support the development of high-quality clinical trials. Additionally, sustained investment in early-career investigators and the creation of structured mentorship pipelines will be critical for cultivating the next generation of leaders in the field. These efforts, taken together, will help transition cardio-rheumatology from a fragmented research niche to a cohesive and impactful domain of cardiovascular science.

This study’s principal strength lies in its comprehensive bibliometric approach, which integrates quantitative analysis of publication output, thematic evolution, and collaboration networks to provide a structured overview of the cardio-rheumatology research landscape. By mapping intellectual contributions, core themes, and emergent areas of inquiry, the analysis offers valuable insights into this evolving field's current state and future direction.

Nevertheless, several limitations must be acknowledged. Bibliometric analyses inherently favor older literature due to citation lag, potentially underrepresenting the impact of more recent, high-quality publications. Additionally, reliance on Scopus-indexed content may exclude relevant research from regional, specialty, or non-indexed journals, particularly those outside the English-language publishing ecosystems. Also, while they are valuable proxies for academic influence, citation metrics do not uniformly reflect clinical relevance, methodological rigor, or translational value and should be interpreted cautiously.

## Conclusions

Cardio-rheumatology is a pivotal inflection point emerging as a clinically relevant and intellectually dynamic field, yet still constrained by insufficient global collaboration and limited interdisciplinary integration. This bibliometric analysis is unique in its synthesis of emerging immunoinflammatory mechanisms, evolving diagnostic strategies, and global health perspectives to propose a cohesive roadmap for the maturation of cardio-rheumatology as a distinct, interdisciplinary field. The growing prominence of cardiac amyloidosis, IL-6-mediated inflammation, and advanced imaging reflects a shifting paradigm toward precision diagnostics and immunomodulatory therapies. The field must prioritize coordinated, inclusive, and globally informed research strategies to meet the increasing clinical demands of autoimmune and inflammatory cardiovascular diseases. Establishing transnational networks, supporting early-career leadership, and fostering innovation will be essential to consolidating cardio-rheumatology as a mature and impactful domain, capable of addressing the complex interface between cardiovascular and rheumatic disease across diverse populations.

## References

[1] Aviña-Zubieta JA, To F, Vostretsova K, De Vera M, Sayre EC, Esdaile JM (2017). Risk of myocardial infarction and stroke in newly diagnosed systemic lupus erythematosus: a general population-based study. Arthritis Care Res (Hoboken).

[2] Gabriel SE (2008). Cardiovascular morbidity and mortality in rheumatoid arthritis. Am J Med.

[3] Gilotra NA, Griffin JM, Pavlovic N (2022). Sarcoidosis-related cardiomyopathy: current knowledge, challenges, and future perspectives state-of-the-art review. J Card Fail.

[4] Patel RP, Parikh R, Gunturu KS, Tariq RZ, Dani SS, Ganatra S, Nohria A (2021). Cardiotoxicity of immune checkpoint inhibitors. Curr Oncol Rep.

[5] Aria M, Cuccurullo C (2017). bibliometrix: an R-tool for comprehensive science mapping analysis. J Informetr.

[6] Birnie DH, Sauer WH, Bogun F (2014). HRS expert consensus statement on the diagnosis and management of arrhythmias associated with cardiac sarcoidosis. Heart Rhythm.

[7] Tschöpe C, Ammirati E, Bozkurt B (2021). Myocarditis and inflammatory cardiomyopathy: current evidence and future directions. Nat Rev Cardiol.

[8] Vakhrusheva O, Smolka C, Gajawada P (2008). Sirt7 increases stress resistance of cardiomyocytes and prevents apoptosis and inflammatory cardiomyopathy in mice. Circ Res.

[9] Blankstein R, Osborne M, Naya M (2014). Cardiac positron emission tomography enhances prognostic assessments of patients with suspected cardiac sarcoidosis. J Am Coll Cardiol.

[10] Frustaci A, Russo MA, Chimenti C (2009). Randomized study on the efficacy of immunosuppressive therapy in patients with virus-negative inflammatory cardiomyopathy: the TIMIC study. Eur Heart J.

[11] Yazaki Y, Isobe M, Hiroe M (2001). Prognostic determinants of long-term survival in Japanese patients with cardiac sarcoidosis treated with prednisone. Am J Cardiol.

[12] Ammirati E, Frigerio M, Adler ED (2020). Management of acute myocarditis and chronic inflammatory cardiomyopathy: an expert consensus document. Circ Heart Fail.

[13] Youssef G, Leung E, Mylonas I (2012). The use of 18F-FDG PET in the diagnosis of cardiac sarcoidosis: a systematic review and metaanalysis including the Ontario experience. J Nucl Med.

[14] Smedema JP, Snoep G, van Kroonenburgh MP, van Geuns RJ, Dassen WR, Gorgels AP, Crijns HJ (2005). Evaluation of the accuracy of gadolinium-enhanced cardiovascular magnetic resonance in the diagnosis of cardiac sarcoidosis. J Am Coll Cardiol.

[15] Kandolin R, Lehtonen J, Airaksinen J (2015). Cardiac sarcoidosis: epidemiology, characteristics, and outcome over 25 years in a nationwide study. Circulation.

[16] (2020). Department of error. Lancet.

[17] Roth GA, Mensah GA, Johnson CO (2020). Global burden of cardiovascular diseases and risk factors, 1990-2019: update from the GBD 2019 study. J Am Coll Cardiol.

[18] Mayosi BM, Flisher AJ, Lalloo UG, Sitas F, Tollman SM, Bradshaw D (2009). The burden of non-communicable diseases in South Africa. Lancet.

[19] Atun R, Knaul FM, Akachi Y, Frenk J (2012). Innovative financing for health: what is truly innovative?. Lancet.

[20] Uzzi B, Mukherjee S, Stringer M, Jones B (2013). Atypical combinations and scientific impact. Science.

[21] Libby P (2002). Inflammation in atherosclerosis. Nature.

[22] Ridker PM, Everett BM, Thuren T (2017). Antiinflammatory therapy with canakinumab for atherosclerotic disease. N Engl J Med.

